# Tuberculosis1Read before the meeting of the Wisconsin Society of Veterinary Graduates, Madison, February 5, 1897.

**Published:** 1897-07

**Authors:** J. J. Oberst

**Affiliations:** Saukville, Wis.


### TUBERCULOSIS.[1]

By J. J. Oberst, M.D.C.,
SAUKVILLE, WIS.

When I promised our Secretary a paper on the subject of tuberculosis I had in mind some experiments which at that time were being conducted by the Wisconsin Experiment Station, and in which I co-operated, hoping that by this time I might be able to report to you some interesting developments with reference to the same.

The experiments were made on a herd of considerable size, consisting of high-bred Guernseys and graded stock, in which the disease was well marked before the suspicion of the owner had become aroused by the developing symptoms.

The chief objects of these experiments were for the purpose of studying the question of restricting tuberculosis by means of proper isolation and hygienic management. Since the former have not been completed, but are to be continued during the coming season, I do not feel justified in publishing any details of the results obtained without the consent of the Experiment Station authorities, save that so far they have been generally satisfactory, considering the existing circumstances, and promise a satisfactory issue, provided they are persisted in for a sufficient length of time.

Since the different phases of this horrible disease are more or less well known to you, I do not deem it necessary to give a lengthy description of the same, since there has been so much written on this subject by most able and prominent men of both the veterinary and medical profession, and with my limited amount of personal experience with this malady I would be unable to add anything particularly new or original.

In view of the foregoing I shall therefore confine my remarks to calling your attention to some most deplorable circumstances in connection with tuberculosis in this State, which are of great importance and demand our earnest consideration. When we read the reports from some of the Eastern States we are astonished at the almost incredible number of cattle, especially of the finer breeds, which are said to be affected and are destroyed as tuberculous. In some of these States more or less stringent laws have been enacted,

[1] Read before the meeting of the Wisconsin Society of Veterinary Graduates, Madison, February 5, 1897.

providing for the destruction of tuberculous animals, with a view of eventually effecting the eradication of this ever-present, universally devastating plague. In some localities the products of animals affected with the disease are strictly prohibited from being used as an article of human food by legal measures, thus affording a much needed protection to the people against infection from that source.

In January last the New York Board of Health adopted an amendment to its sanitary code declaring tuberculosis an infectious and communicable disease, dangerous to the public health. Reports of all cases hereafter must be made to the sanitary bureau in writing, when the same will be treated in the same manner as cases of diphtheria and scarlet fever. If similar actions were adopted all over the United States with reference to human and bovine tuberculosis, and the same were strictly and conscientiously carried into effect, the number of deaths caused by the destructive action of the tubercle bacillus would become rapidly diminished and the further dissemination of this most dangerous of all contagious diseases as far as practicable rendered an impossibility.

I say the most dangerous of all contagious diseases because it affects both man and beast alike, and is frequently transmissible from animal to man, as where animals having the disease in an advanced stage are kept in close quarters, devoid of sunlight or a free access of fresh air, where the drainage is but imperfect or perhaps entirely wanting, thus causing the liquid portions of manure and other decaying substance to accumulate, and in their process of decomposition give off noxious and repulsive odors, which in their turn offer a favorable medium for the proliferation of all disease-producing germs. If anyone should attempt to enter such a barn after the same had been closed up for some time, during which the atmosphere therein had become impregnated with the germs of disease, he would be almost certain to become infected, especially if he should be suffering from any inflammatory disease of the air-passages.

That a frequent source of infection lies in the indiscriminate use of milk from tuberculous cows goes without saying, as there are numerous cases on record where the tubercle bacilli have been discovered in the milk from cows, which, to all external appearances, seemed to be in perfect health and condition; also in the use of meat from tuberculous animals for human food we cannot fail to recognize the danger of infection.

Transmission of the disease from man to animals may also occur where persons suffering from pulmonary tuberculosis in an advanced stage are attending the animals, and while thus engaged carelessly allow the tubercular matter coughed up from their diseased lungs to fall upon the food, and thus be taken into the alimentary tract of the animals while eating; or the expectorated matter may fall upon the floor or other places, where it becomes dry and pulverized, when afterward, through some mechanical cause, it is stirred up into the air in the form of dust, and the germs thus liberated, if in sufficient proximity to the animal, are during the inspiratory act taken into the air-passages of the same, where they become lodged and start the disease.

That tuberculosis has not yet gained such an extensive distribution in this State as in the East I am happy to acknowledge, yet I do believe that if thorough investigations were made by proper and competent investigators the number of animals found to be affected with the disease would greatly exceed the general expectation.

To my knowledge there have been no provisions made in the laws of this State requiring the destruction of animals suffering from this disease in an advanced form, so as to exclude any probable chance of recovery, nor to provide for the quarantining of those less dangerously diseased; nor is any considerable amount of protection afforded to the people against infection through prohibiting the use of products from tuberculous animals as an article of food. Under such inadequate legislation the disease is offered every opportunity for its rapid dissemination, as animals may be bought and sold from herds in which the disease exists. Even though the animals referred to may, to all external appearances, be in a state of perfect health, they may yet be affected with the disease in the incipient stage, and by such a transaction carry it with them into another previously healthy herd, and perhaps cause members of that herd to become diseased before any symptoms develop that attract the attention of the unsuspecting owner.

That numerous cases of infection to the human family in this State are caused by the indiscriminate use of meats from tuberculous animals is a deplorable fact which cannot leave any room for doubt when we consider the criminal indifference with which such diseased meats are prepared and sold for human consumption. This statement may perhaps appear to you to be greatly exaggerated; but kindly allow me to relate to you a little incident which occurred during the past summer, and you will at once understand my reason for taking this view of the subject. During the first part of August last, more out of curiosity than suspicion, I was requested by Mr. A. to inspect some casings (bovine intestines prepared for the purpose of being used for sausage coverings), which, as he said, were studded with little nodules. On examination I found nearly all the contents of a 200-pound barrel to be affected with pearl-disease. I informed Mr. A. accordingly, and cautioned him against utilizing any of them for any purpose, which advice was observed. A specimen from the same intestines was sent to Prof. H. L. Russell, of Madison, for microscopic examination, which later resulted in the verification of my statement.

On the same occasion the gentleman informed me that while in a large slaughtering and sausage manufacturing establishment in one of the principal cities of this State, where he had been employed for several successive years, they had killed a large number of cattle in which the intestines, peritoneum, and pleuræ were covered with tubercular growths, and that they had often found abscesses and pus-cavities in the various internal organs, especially the lungs, but that in no case was the carcass rejected, but the whole was utilized and sold chiefly in the form of sausages.

Other persons who had lived in a large city informed me that they had frequently observed some of the glandular organs of animals hanging in meat-markets, and had even themselves bought and consumed parts of them after the remaining portions, which were filled with abscesses of varying sizes, had been cut away and thrown into the swill-box, not having any idea of the danger to which they had thereby exposed themselves and others.

Should not we, in the light of such horrifying evidence, as true veterinarians and preservers of the public health, which in fact the true veterinarian is, join in a united and untiring effort to strive toward obtaining proper legislation by which we may be enabled to call an energetic halt and effectually banish from our midst this unmerciful slayer of both man and animal? I do not wish to be understood as one in favor of slaughtering tuberculous animals by the wholesale, as I believe that an animal having the disease in the incipient stage has every chance to recover and become practically cured, provided it receives proper care in regard to food and drink and general hygienic management. All animals which have become so badly diseased as to be beyond recovery should be absolutely destroyed, those less dangerously diseased put under strict quarantine regulations. The products of tuberculous animals should be strictly prohibited as an article of food unless they are rendered sterile, so as to remove all probable danger of infection either to the human or animals. The local boards of health should be composed of members having sufficient knowledge of contagious diseases to enable them at least to distinguish the most prominent symptoms of such diseases, instead of, as is frequently the case, having no knowledge fitting them for the office to which they have been elected, and consequently being unable to discharge their duties in a proper manner. The right to order and impose a quarantine on tuberculous animals should be extended to the local boards of health, and none but legally qualified and duly registered veterinarians should be allowed to make any tests or in any other way attempt the management of tuberculous animals. Not until we are aided by legislation will we be able to succeed in our effort to restrict the spread of this disease. The present law leaves too much chance for being disobeyed, and if not altered in time we may expect within a few years the same percentage of tuberculosis among both man and animals in this State, as is at present reported from the East. Therefore, let us work in time, and not remain idle until the enemy appears in such overwhelming numbers as to greatly diminish our opportunity to gain a glorious victory.

